# Rank-based methods for modeling dependence between loss triangles

**DOI:** 10.1007/s13385-016-0134-y

**Published:** 2016-07-11

**Authors:** Marie-Pier Côté, Christian Genest, Anas Abdallah

**Affiliations:** 1 Department of Mathematics and Statistics, McGill University, 805, rue Sherbrooke Ouest, Montréal, Québec H3A 0B9 Canada; 2 École d’actuariat, Université Laval, 1045, avenue de la Médecine, Québec, Québec G1V 0A6 Canada

**Keywords:** Capital allocation, Copula, GLM, Hierarchical modeling, Nested Archimedean copulas, Parametric bootstrap, Rank-based estimation, Risk aggregation, Run-off triangles

## Abstract

In order to determine the risk capital for their aggregate portfolio, property and casualty insurance companies must fit a multivariate model to the loss triangle data relating to each of their lines of business. As an inadequate choice of dependence structure may have an undesirable effect on reserve estimation, a two-stage inference strategy is proposed in this paper to assist with model selection and validation. Generalized linear models are first fitted to the margins. Standardized residuals from these models are then linked through a copula selected and validated using rank-based methods. The approach is illustrated with data from six lines of business of a large Canadian insurance company for which two hierarchical dependence models are considered, i.e., a fully nested Archimedean copula structure and a copula-based risk aggregation model.

## Introduction

In Canada, the Own Risk Solvency and Assessment (ORSA) guideline from the Office of the Superintendent of Financial Institutions (OSFI) requires that insurance companies set internal targets for risk capital that are tailored to their consolidated operations. In order to relate risk to capital and consider their operations as a whole, insurers are encouraged to develop internal models for the aggregation of dependent risks. Similar regulations exist in many countries worldwide.

To comply with regulatory standards, property and casualty insurance companies have to hold reserves and risk capital relating to losses that are incurred but not yet paid. For each line of business, payments relating to past claims are usually structured in a run-off triangle arranged to rows according to the accident years, and to columns according to the development periods, i.e., the years since the accident occurred. In order to determine a reserve, one must forecast the payments that these ongoing claims will induce in future years, i.e., one must extend each triangle to a rectangle by predicting the missing entries.

Several nonparametric approaches are available for developing claims in a run-off triangle, most notably the chain-ladder method. In order to account for the dependence between triangles, multivariate extensions of this technique have been proposed, e.g., in [7, 28, 31, 34, 41]. These techniques account for dependence in the computation of reserves and their prediction errors but they do not provide the predictive distribution needed to obtain risk measures such as Value-at-Risk (VaR) or Tail Value-at-Risk (TVaR). Their use in the determination of risk capital is therefore limited.

Parametric approaches leading to the distribution of unpaid losses have been considered, e.g., in [1, 8, 12, 29, 36, 37]. Models investigated in these articles incorporate dependence between lines of business and/or within calendar years of a line of business through Gaussian, Archimedean or Hierarchical Archimedean copulas. In these papers, the total reserve estimate in the presence of dependence is not equal to the sum of the marginal reserves estimated assuming independence. This is a by-product of the joint estimation of the marginal and dependence parameters, which relies heavily on the choice of multivariate model for the run-off triangles. An inadequate choice of dependence structure may then have a large, undesirable effect on the estimation of the reserves. This is particularly worrying given that this choice is typically based on very few data points (e.g., 55 observations for 10 accident years and 10 development periods). Tools are thus needed for assessing the dependence between run-off triangles and selecting an appropriate model.

In this paper, we address this inferential issue within the context of a multivariate extension of the pairwise model of [37], where the dependence between corresponding cells of different run-off triangles is described by a copula. We propose to use an alternative two-stage inference strategy, in which generalized linear models (GLMs) are first fitted to the margins, thereby fixing the estimates of the reserves. In the second step, standardized residuals from those models are linked through a dependence structure estimated using rank-based methods. This general approach has a long history in the copula modeling literature; see, e.g., [14] or [17] for reviews. When dealing with identically distributed data, rank-based methods are well-established tools for selecting, estimating and validating copulas. To our knowledge, however, these techniques have never been applied to run-off triangles.

To illustrate the proposed approach, we consider run-off triangles for six portfolios from a large Canadian property and casualty insurance company. These data are described in Sect. 2 and appended. In Sect. 2.1, GLMs with log-normal and Gamma distributions are fitted to the individual portfolios, and the properties of these two parametric families are exploited in Sect. 2.2 to define residuals that are suitable for a dependence analysis through ranks. Two different hierarchical approaches are then explored for modeling the dependence between the lines of business.

In Sect. 3, a nested Archimedean copula model is fitted, along the same lines as [1]. As this model imposes many constraints on the dependence structure and the choice of copulas, a more flexible approach considered in [4, 11] is implemented in Sect. 4. Risk capital calculations and allocations for the two models are compared in Sect. 5, and Sect. 6 summarizes the pros and cons of these approaches. Appendix 1 contains density calculations for the nested Archimedean copula model, and the data (up to a multiplicative factor for confidentiality purposes) are provided in Appendix 2, along with parameter estimates of the marginal GLMs.

## Data

The run-off triangle data considered in this paper are from a large Canadian property and casualty insurance company. They consist of the cumulative paid losses and net earned premiums for six lines of automobile and home insurance business. Tables 13, 14, 15, 16, 17 and 18 in Appendix 2 show the paid losses for accident years 2003–12 inclusively for each of the six lines of business developed over at most ten years. To preserve confidentiality, all figures were multiplied by a constant. However, this is inconsequential because in order to account for the volume of business, the analysis focuses on the paid loss ratios, i.e., the payments divided by the net earned premiums.

Table 1 gives a descriptive summary of each line of business (LOB). There are five run-off triangles of personal and commercial auto lines with accident benefits and bodily injury coverages from three regions (Atlantic, Ontario and the West). Atlantic Canada consists of New Brunswick, Nova Scotia, Prince Edward Island and Newfoundland/Labrador; the West comprises Manitoba, Saskatchewan, Alberta, British Columbia, Northwest Territories, Yukon, and Nunavut. Given that Québec has a public plan for this section of auto insurance, business for that province is included only in the sixth triangle, which comprises the company’s country-wide Liability personal and commercial home insurance.

**Table 1:** Descriptive summary of six lines of business for a Canadian insurance company

LOB	Region	Product	Coverage
1	Atlantic	Auto	Bodily injury
2	Ontario	Auto	Bodily injury
3	West	Auto	Bodily injury
4	Ontario	Auto	Accident benefits excluding disability income
5	Ontario	Auto	Accident benefits: disability income only
6	Country-wide	Home	Liability

Bodily injury (BI) coverage provides compensation to the insured if the latter is injured or killed through the fault of a motorist who has no insurance, or by an unidentified vehicle. The accident benefits (AB) coverage provides compensation, regardless of fault, if a driver, passenger, or pedestrian suffers injury or death in an automobile collision. Disability income is an insurance product that provides supplementary income when the accident results in a disability that prevents the insured from working at his/her regular employment. For this reason, AB disability income is considered separately from other AB. Finally, liability insurance covers an insured for his/her legal liability for injuries or damage to others.

### Marginal GLMs for incremental loss ratios

For LOB $\ell \in \{ 1,\ldots ,6 \}$, denote by $Y_{ij}^{(\ell )}$ the incremental payment for the *i*th accident year and the *j*th development period, where $i,j \in \{ 1,\ldots ,10\}$. Given that the earned premiums $p_{i}^{(\ell )}$ vary with accident year *i* and line of business $\ell$, it is convenient to model the loss ratios, defined by

$$\begin{aligned} X_{ij}^{(\ell )} = Y_{ij}^{(\ell )}/p_{i}^{(\ell )}. \end{aligned}$$

**Fig. 1:**
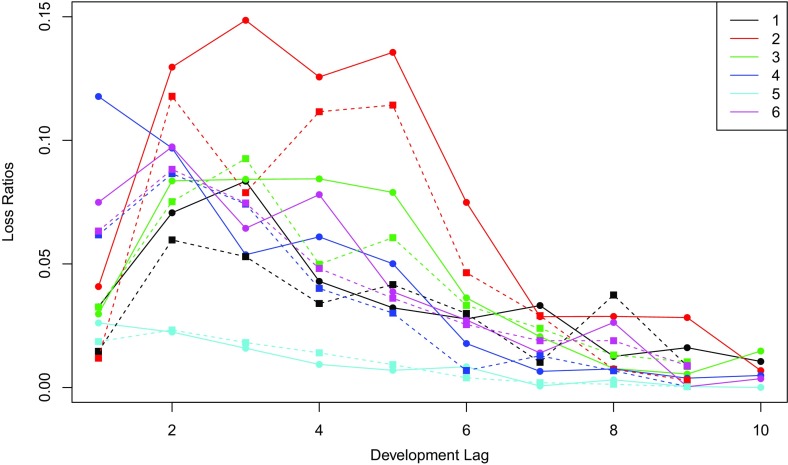
Loss ratios for years 2003 (*solid line*) and 2004 (*dashed line*) in function of the development lag for the six lines of business

In Fig. 1, loss ratios $X_{ij}^{(\ell )}$ for $i=1,2$, $j=1,\ldots , 11-i$ and $\ell =1,\ldots ,6$ are shown. It is clear from the graph that the loss ratio depends on the development lag for every portfolio. By comparing the solid and dashed lines of the same color, one can also see that the accident year has an impact. In order to capture these patterns, we consider a regression model with two explanatory variables, i.e., accident year and development period. This is in line with the classical chain-ladder approach.

For LOB $\ell \in \{ 1,\ldots ,6 \}$, let $\kappa _{i}^{(\ell )}$ be the effect of accident year $i \in \{1,\ldots , 10\}$ and $\lambda _{j}^{(\ell )}$ be the effect of development period $j \in \{1,\ldots ,10\}$. The systematic component for the $\ell$th line of business can then be written as

$$\begin{aligned} \eta _{ij}^{(\ell )} = \zeta ^{(\ell )} + \kappa _{i}^{(\ell )} + \lambda _{j}^{(\ell )}, \end{aligned}$$

where $\zeta ^{(\ell )}$ is the intercept, and for parameter identification, we set $\kappa _{1}^{(\ell )} = \lambda _{1}^{(\ell )} = 0$. There is no interaction term in this model, i.e., it is assumed that the effect of a given development period does not vary by accident year. While this assumption is hard to check, it is required to ensure that all parameters can be estimated from the 55 observations available.

In their analysis of dependent loss triangles using copulas, Shi and Frees [37] use the log-normal and Gamma distributions for incremental claims. Their justification applies here as well. Following these authors, we consider the link

$$\begin{aligned} \mu _{ij}^{(\ell )}=\eta _{ij}^{(\ell )} \end{aligned}$$

for a log-normal distribution with mean $\mu _{ij}^{(\ell )}$ and standard deviation $\sigma ^{(\ell )}$ on the log scale. For the Gamma distribution, however, we use the exponential link instead of the canonical inverse link in order to enforce positive means. When the Gamma distribution is selected, therefore, its scale and shape parameters are respectively denoted by $\beta _{ij}^{(\ell )}$ and $\alpha ^{(\ell )}$, and it is assumed that

$$\begin{aligned} \beta _{ij}^{(\ell )}=\exp (\eta _{ij}^{(\ell )})/\alpha ^{(\ell )}. \end{aligned}$$

Log-normal and Gamma distributions were fitted to all lines of business by the method of maximum likelihood. Table 2 shows the corresponding values of the Akaike information criterion (AIC) and the Bayesian information criterion (BIC). These criteria suggest the choice of the log-normal distribution for the first line of business and the Gamma distribution for all others. These choices of models are confirmed by the Kolmogorov–Smirnov goodness-of-fit test, whose *p*-values are also given in Table 2. No model is rejected at the 1 % level. Q–Q plots (not shown) of standardized residuals (defined below) provide visual confirmation that the selected models are adequate, although the fit for LOB 6 is borderline.

**Table 2:** Fit statistics and goodness-of-fit test of marginals

LOB	AIC		BIC		*p*-value of the Kolmogorov–Smirnov test
	Log-normal	Gamma	Log-normal	Gamma	
1	−294	−291	−254	−251	0.886
2	−266	−270	−226	−230	0.643
3	−323	−324	−283	−283	0.397
4	−272	−276	−232	−236	0.135
5	−441	−444	−401	−404	0.478
6	−259	−267	−219	−226	0.019

Parameter estimates of the fitted models are given in Appendix 2 along with their standard errors. Using these values, one can estimate the total reserve of the portfolio by

$$\begin{aligned} \sum _{\ell =1}^{6}\sum _{i=2}^{10}\sum _{j=10-i+2}^{10}p_{i}^{(\ell )}\mathrm{E}(X_{ij}^{(\ell )}), \end{aligned}$$

where $\mathrm{E}(X_{ij}^{(\ell )})$ is the projected unpaid loss ratio, and $p_{i}^{(\ell )}$ is the premiums earned in the corresponding accident year *i*. For $\ell =1$, we have

$$\begin{aligned} \mathrm{E}(X_{ij}^{(1)}) = \exp \{\hat{\mu }_{ij}^{(1)}+(\hat{\sigma }^{(1)})^{2}/2\}, \end{aligned}$$

while for $$\ell >1$$, $$\mathrm{E}(X_{ij}^{(\ell )}) = \hat{\beta }_{ij}^{(\ell )}\hat{\alpha }^{(\ell )}$$. The estimated reserves of the six lines of business are given at the bottom of Table 19 in Appendix 2, along with those derived from the chain-ladder method, which is the industry’s benchmark. The two methods lead to similar results and total reserve estimates of $438,088 and $453,686, respectively.

### Exploratory dependence analysis

One would expect intuitively that the AB, BI and liability claim payments are associated, as these coverages all involve compensation for injuries or damage to the insured or to others. One may also wonder whether there exist interactions between portfolios across regions. In order to account for such dependencies between $d\ge 2$ triangles, Shi and Frees [37] propose to link the marginal GLMs through a copula. This approach involves expressing the joint distribution of the loss ratios in the form

$$\begin{aligned} \Pr (X_{ij}^{(1)}\le x_{ij}^{(1)},\ldots ,X_{ij}^{(d)}\le x_{ij}^{(d)}) = C\{ \Pr (X_{ij}^{(1)}\le x_{ij}^{(1)}), \ldots , \Pr (X_{ij}^{(d)}\le x_{ij}^{(d)})\}, \end{aligned}$$

where *C* is a *d*-variate cumulative distribution function with uniform margins on (0, 1).

**Fig. 2:**
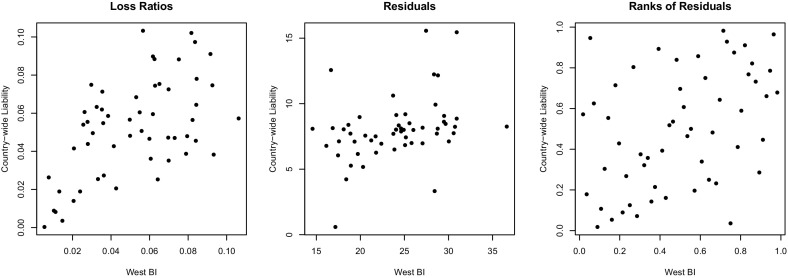
Loss ratios (*left*), residuals (*middle*) and standardized ranks of the latter (*right*) for LOBs 3 and 6

In order to select a copula *C* that appropriately reflects the dependence in the data, it is best to rely on rank-based techniques as they allow to separate the effect of the marginals from the dependence structure [14, 17].

To illustrate this point, consider first the graph displayed in the left panel of Fig. 2, which shows a scatter plot of the pairs $(X_{ij}^{(3)},X_{ij}^{(6)})$ with $i,j\in \{1,\ldots ,10\}$ and $j\le i$. This graph suggests a strong, positive dependence between BI in Western Canada and country-wide liability; in particular, the Pearson correlation is 0.56. However, the pattern of points on this graph is induced by the systematic effects of the development lags and accident years. For example, the seven points in the lower left corner of the graph all correspond to development years 7–10. As these effects are already accounted for by the marginal GLMs, this graph is uninformative (not to say misleading) for the selection of *C*.

To get insight into the dependence structure, it is more relevant to consider the residuals from the GLMs. For LOB 1, (standardized) residuals of the log-normal regression model can be defined, for all $i,j\in \{1,\ldots ,10\}$ and $j\le i$, as

$$\begin{aligned} \varepsilon ^{(1)}_{ij} = \{\ln (X^{(1)}_{ij})-\hat{\mu }_{ij}^{(1)} \}/\hat{\sigma }^{(1)}, \end{aligned}$$

while for LOB $\ell \in \{2,\ldots ,6\}$, the fact that Gamma regression models were used leads to set

$$\begin{aligned} \varepsilon _{ij}^{(\ell )}=X_{ij}^{(\ell )}/\hat{\beta }_{ij}^{(\ell )}. \end{aligned}$$

In this fashion, the vectors $(\varepsilon ^{(1)}_{ij},\ldots ,\varepsilon ^{(6)}_{ij})$ with $i,j\in \{1,\ldots ,10\}$ and $j\le i$ form a pseudo-random sample from a distribution with copula *C* and margins approximately $\mathscr{N}(0,1)$ for $\ell =1$ and $\mathscr{G}(\hat{\alpha }^{(\ell )},1)$, for $\ell \in \{ 2,\ldots ,6\}$.

As an illustration, the middle panel of Fig. 2 shows a scatter plot of the pairs $(\varepsilon _{ij}^{(3)},\varepsilon _{ij}^{(6)})$. This graph suggests a form of positive dependence (Pearson’s correlation is 0.34), but the message is blurred by the effect of the Gamma marginals. As the goal is to select the copula *C*, which does not depend on the margins, it is preferable to plot the pairs of normalized ranks, as in the right panel of Fig. 2. For arbitrary $i,j\in \{1,\ldots ,10\}$ and $j\le i$, the standardized rank of residual $\varepsilon _{ij}^{(\ell )}$ is defined by

$$\begin{aligned} R_{ij}^{(\ell )} = \frac{1}{55+1} \sum _{i^*=1}^{10}\sum _{j^*=1}^{11-i^*} \mathbf {1}(\varepsilon _{i^*j^*}^{(\ell )} \le \varepsilon _{ij}^{(\ell )}), \end{aligned}$$

where, in general, $\mathbf {1}(A)$ is the indicator function of the set *A* and the division by 56 rather than 55 is to ensure that all standardized ranks are strictly comprised between 0 and 1.

Let $C_n$ be the empirical distribution function of the vectors $(R_{ij}^{(1)},\ldots ,R_{ij}^{(d)})$, with $i,j\in \{1,\ldots ,10\}$ and $j\le i$. It can be shown, under suitable conditions on the underlying copula *C*, that $C_n$ is a consistent estimator thereof. Accordingly, the vectors of standardized ranks, which form the support of $C_n$, are a reliable tool for copula selection, fitting and validation. In particular, all rank-based tests of bivariate or multivariate independence are based on $C_n$.

For example, the right panel of Fig. 2 shows the pairs of standardized ranks associated with the residuals from the West BI and the country-wide liability coverages. One can see from this graph that there is a residual dependence between these two portfolios. In particular, the correlation between these pairs is 0.40; this rank-based correlation is a consistent estimate of Spearman’s $\rho$. Alternative copula-based measures of association between two variables are Kendall’s $\tau$ and van der Waerden’s coefficient $\Upsilon$. Thus one can test the null hypothesis of bivariate independence by checking whether the empirical values of these coefficients are significantly different from 0; see, e.g., [23]. Table 3 gives estimates of $\rho$, $\tau$ and $\Upsilon$ for the pair $(\varepsilon ^{(3)},\varepsilon ^{(6)})$, along with the *p*-values of the corresponding tests; the null hypothesis of independence is rejected at the 1 % level in all cases.

**Table 3:** Nonparametric tests of independence

Kendall’s test		Spearman’s test		van der Waerden test	
$\hat{\tau }$	*p*-value	$\hat{\rho }$	*p*-value	$\hat{\Upsilon }$	*p*-value
0.29	0.0021	0.40	0.0023	18.27	0.0055

**Table 4:** Empirical values of Kendall’s $\tau$ for all pairs in the portfolio

	ε^(1)^	ε^(2)^	ε^(3)^	ε^(4)^	ε^(5)^	ε^(6)^
ε^(1)^	1.000	0.115	0.024	**−0.061**	0.014	0.076
ε^(2)^	0.115	1.000	**−0.331**	**0.244**	**0.209**	−0.090
ε^(3)^	0.024	**−0.331**	1.000	0.040	−0.079	**0.285**
ε^(4)^	−0.061	**0.244**	0.040	1.000	**0.200**	0.030
ε^(5)^	0.014	**0.209**	−0.079	**0.200**	1.000	0.046
ε^(6)^	0.076	−0.090	**0.285**	0.030	0.046	1.000

Bold values indicate significantly different from 0 at the 5 % level in a single pairwise test

The null hypothesis of multivariate independence between the six LOBs can also be assessed globally using rank tests based on *d*-variate generalizations of $\rho$, $\tau$ or $\Upsilon$. In particular, the *d*-variate version of Kendall’s $\tau$ is given, e.g., in [18], by

$$\begin{aligned} \tau _{d,n}=\frac{1}{2^{d-1}-1}\left\{ -1+\frac{2^d}{n(n-1)} \sum _{(i,j) \ne (i^*,j^*)}\mathbf {1}\left( \varepsilon _{i^*j^*}^{(1)}\le \varepsilon _{ij}^{(1)},\ldots ,\varepsilon _{i^*j^*}^{(d)}\le \varepsilon _{ij}^{(d)}\right) \right\} =0.035. \end{aligned}$$

Under the hypothesis of multivariate independence, $\tau _{d,n}$ has mean 0, finite sample variance

$$\begin{aligned} \mathrm{var}(\tau _{d,n})=\frac{n(2^{2d+1}+2^{d+1}-4\times 3^d)+3^d(2^d+6)-2^{d+2}(2^d+1)}{3^d(2^{d-1}-1)^2n(n-1)}=1.59\times 10^{-4}, \end{aligned}$$

and its distribution is asymptotically Gaussian. The approximate *p*-value of the test is $0.53~\%$, suggesting that the residuals are dependent. The most dependent pairs of variables can be identified from Table 4, where all values of $\tau _{2,n}$ are displayed. Values shown in bold are those that would be significantly different from 0 at the 5 % level in a single pairwise test. Although this level must be interpreted with care due to the multiple comparison issue, the two largest values in Table 4 are still significantly different from 0 at the global 5 % level even when the very conservative Bonferroni correction is applied.

**Table 5:** Parameter estimates and goodness-of-fit test *p*-value

Copula	Parameter	Standard deviation	*p*-value
Clayton	0.584	0.194	0.0804
Frank	2.804	0.836	0.7557
Plackett	3.777	1.426	0.7747
$t_{2}$	0.375	0.155	0.2323

Given the presence of dependence, the challenge is then to select a copula that best reflects the association between the variables. Many parametric families of copulas are available; see, e.g., [27] or [30] for the definition and properties of the Clayton, Frank, Plackett and *t* copula families used subsequently. Given a class $\mathscr{C}=\{C_\theta :\theta \in \Theta \}$ of *d*-dimensional copulas, a rank-based estimate $\hat{\theta }$ of the dependence parameter $\theta$ can be obtained from loss-triangle data by maximizing the pseudo log-likelihood

$$\begin{aligned} \mathscr{L}(\theta )=\sum _{i=1}^{10}\sum _{j=1}^{11-i}\ln \{c_\theta (R^{(1)}_{ij},\ldots , R^{(d)}_{ij})\}, \end{aligned}$$

where $c_\theta$ is the density of $C_\theta$. The consistency and asymptotic normality of estimators of this type was established in [15] under broad regularity conditions. The adequacy of the class $\mathscr{C}$ can then be tested using the Cramér–von Mises statistic defined by

$$\begin{aligned} S_n=\int _{[0,1]^d} \left\{ C_n(u_1,\ldots ,u_d)-C_{\hat{\theta }}(u_1,\ldots ,u_d)\right\} ^2\mathrm{d}u_1\cdots \mathrm{d}u_d. \end{aligned}$$

The *p*-value of a test of the hypothesis $\mathscr{H}_0: C\in \mathscr{C}$ based on the statistic $S_n$ can be computed via a parametric bootstrap procedure described in [19]. Both the estimation and the goodness-of-fit procedures are available in the R package copula. For illustration, Table 5 shows the parameter estimates, standard deviation and the *p*-value of the goodness-of-fit test for four copula families fitted to the pairs of residuals $(\varepsilon ^{(3)},\varepsilon ^{(6)})$ from the West BI and country-wide Liability triangles. This suggests that the Clayton copula would be a poor choice for these data; given the small sample size, however, it does not seem possible to discriminate between the other three copula families on the basis of $S_n$.

**Fig. 3:**
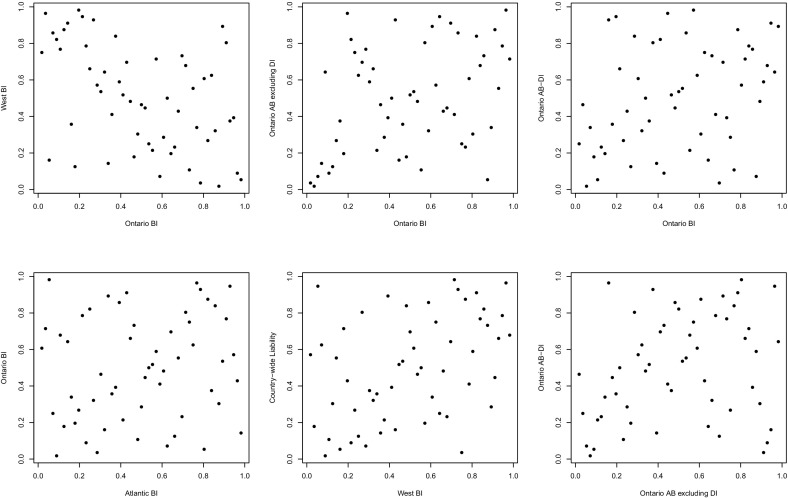
Scatter plot of residuals between different LOBs

This model selection, fitting and validation procedure is standard and straightforward to implement in two dimensions. However, the canonical *d*-variate generalizations of bivariate copulas typically lack flexibility: either they are exchangeable and/or their lower-dimensional margins are all of the same type. With six lines of business, these assumptions may be too restrictive. As one can see in Fig. 3, different pairs of residuals exhibit different types of association; this is also confirmed by the values of Kendall’s $\tau$ reported earlier in Table 4. In particular, Ontario LOBs exhibit positive dependence, while the BI coverages for Ontario and the West are negatively associated.

The fact that many variables are positively dependent is due in part to exogenous common factors such as inflation and interest rates. Furthermore, strategic decisions can impact several portfolios, e.g., the acceleration of payments on all lines of the liability insurance sector could induce some dependence between West BI and country-wide liability. At a more basic level, the positive association between Ontario AB and BI can be explained by the fact that the same accident will often arise in both coverages. Finally, jurisprudence can play a role. For example, reforms were engaged in the Atlantic region to control BI costs; this may explain why LOB 1 is seemingly independent from all other lines of business.

## Nested Archimedean copula model

Nesting Archimedean copulas is a popular way of constructing non-exchangeable multivariate dependence models. This approach, originally proposed in [24], was further investigated, e.g., in [13, 33, 40]. In the reserving literature, Abdallah et al. [1] exploited nested Archimedean copulas to model the dependence between two run-off triangles. In what follows, this approach is extended to higher dimensions using a specific structure called fully nested Archimedean copulas.

Following [16] or [30], a bivariate copula is said to be Archimedean with generator $\varphi _1:(0,1]\rightarrow [0,\infty )$ if it can be expressed, for all $(u_1,u_2) \in (0,1)^2$, in the form

$$\begin{aligned} C_1(u_1,u_2)=\varphi _1^{-1}\{\varphi _1(u_1)+\varphi _1(u_2)\}, \end{aligned}$$

where $\varphi _1$ is convex, decreasing and such that $\varphi _1(1)=0$. More generally, a $(d+1)$-variate copula $C_d$ is said to be a fully nested Archimedean copula with generators $\varphi _{1},\ldots ,\varphi _{d}$ if it is defined recursively for all $(u_1,\ldots ,u_{d+1})\in (0,1)^{d+1}$, by

$$\begin{aligned} \begin{array}{lll} C_2(u_1,u_2,u_{3})&{}=&{}\varphi ^{-1}_{2}[ \varphi _{2}(u_{3})+\varphi _{2}\{C_{1}(u_1,u_2)\}], \\ \quad \vdots &{}=&{}\quad \vdots \\ C_d(u_1,\ldots ,u_{d+1})&{}=&{}\varphi ^{-1}_{d}[ \varphi _{d}(u_{d+1})+\varphi _{d}\{C_{d-1}(u_1,\ldots ,u_{d})\}].\\ \end{array} \end{aligned}$$

As shown in [26], $C_d$ is a copula when the following conditions hold:

1. $\varphi _{1}^{-1},\ldots ,\varphi _{d}^{-1}$ are completely monotone, i.e., Laplace transforms;
2. $\varphi _{k+1}\circ \varphi _{k}^{-1}$ has completely monotone derivatives for all $k\in \{1,\ldots ,d-1\}$.

This model is such that if $(U_1,\ldots ,U_{d+1})$ is distributed as $C_d$, the copula linking variables $U_j$ and $U_k$ is Archimedean with generator $\varphi _{k-1}$ for all $j<k$. Because of condition (2), one must also have

1 $$\begin{aligned} \tau (U_k,U_\ell )\le \tau (U_i,U_j), \quad i<j<\ell , \quad k<\ell . \end{aligned}$$

Algorithms for generating data from $C_d$ were given in [21, 26]. Hofert and Mächler [22] also wrote the R package nacopula (now merged into copula) that can be used to simulate from fully nested Archimedean copulas in any dimension.

**Fig. 4:**
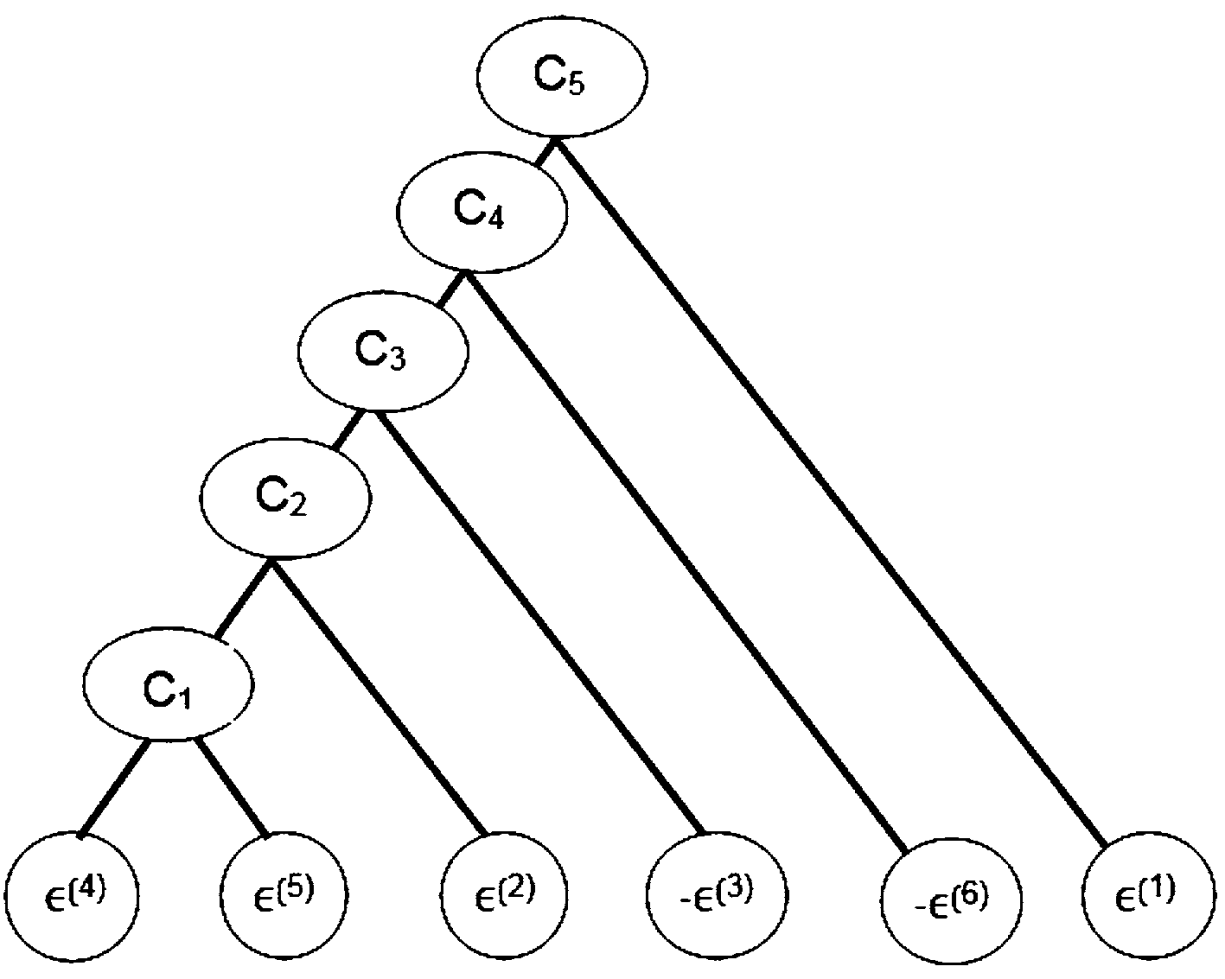
Tree structure for the fully nested Archimedean copula model

Figure 4 depicts the fully nested Archimedean structure used to model the dependence between the residuals of the six lines of business. In this structure, copula $C_1$ links the two components of the Ontario AB coverage. Their dependence with Ontario BI coverage is then incorporated at level 2. The West BI and the country-wide Liability coverages are then included at levels 3 and 4, respectively. Anti-ranks (i.e., the ranks of the negative residuals) had to be used at levels 3 and 4, because of the constraints imposed by (1) and the fact that the residuals for LOB 3 are negatively associated with LOB 2 and positively associated with LOB 6. Finally, the Atlantic BI coverage was included at the last step given its apparent lack of dependence with the other lines of business. This overall structure is in accordance with ratemaking practices, as the rating is typically performed on a territorial basis. One may thus expect the dependence between lines of business to be larger when they are from the same region than when they are not.

In what follows, it is assumed that for each $k \in \{ 1, \ldots , 5\}$ and all $t \in (0,1)$,

$$\begin{aligned} \varphi _k (t) = -\ln \left( \frac{e^{-t\theta _k}-1}{e^{-\theta _k}-1}\right) \end{aligned}$$

for some $\theta _k \in \mathbb {R}$. In other words, the nested copulas are taken to be from the Frank family, which spans all degrees of dependence between $-1$ and 1, as measured by Kendall’s $\tau$. A rank-based estimate $\hat{\varvec{\theta }}$ of the vector $\varvec{\theta }= (\theta _1,\ldots ,\theta _5)$ characterizing the dependence structure is then obtained by maximizing the pseudo-likelihood function

$$ {\mathscr{L}}(\theta ) = \sum\limits_{{i = 1}}^{{10}} {\sum\limits_{{j = 1}}^{{11 - i}} {\ln } \left\{ {c\left( {R_{{ij}}^{{(4)}} ,R_{{ij}}^{{(5)}} ,R_{{ij}}^{{(2)}} ,1 - R_{{ij}}^{{(3)}} ,1 - R_{{ij}}^{{(6)}} ,R_{{ij}}^{{(1)}} ;\theta } \right)} \right\},}  $$

where *c* is the density of the fully nested Archimedean copula. As shown in Appendix 1, the evaluation of this density is straightforward but computationally intensive in high dimensions. Therefore, due to evidence that residuals for LOB 1 are independent from residuals for other LOBs, $\theta _5$ was set equal to 0.

**Fig. 5:**
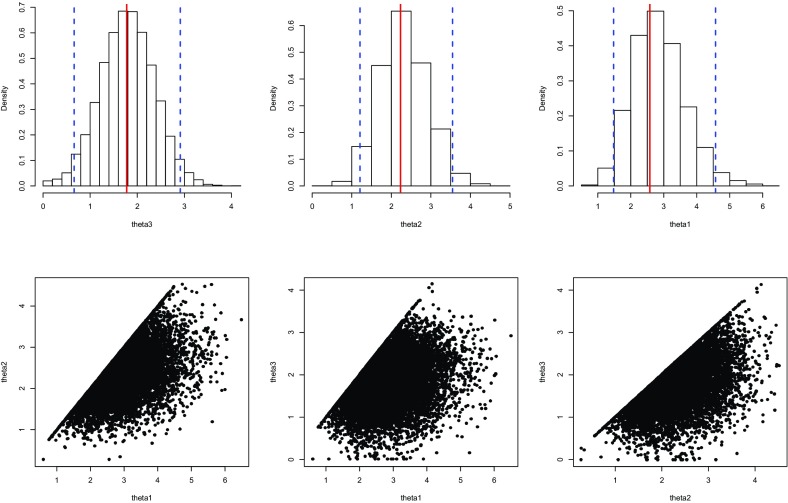
Fully nested Archimedean copula model: histograms of bootstrap parameters with 95 % confidence interval (*top row*) and scatter plots of bootstrap replications (*bottom row*)

The maximization of the pseudo-likelihood for the model with four levels leads to the parameter estimate $\hat{\theta }=(2.693,2.354,1.782,0.867)$. However, a 95 % confidence interval for $\theta _4$ based on 1000 bootstrap replicates includes 0, which corresponds to independence in the Frank copula family. Accordingly, the dependence is significant only in the first three levels of the hierarchy. The parameters of the reduced model with $\theta _4 = \theta _5 = 0$ were estimated once again by the maximum pseudo-likelihood method. This led to $\hat{\theta }=(2.577,2.233,1.776)$, whose components are all significantly different from 0.

Figure 5 shows the approximate distribution of $\hat{\theta }_3$ (left), $\hat{\theta }_2$ (middle), and $\hat{\theta }_1$ (right) based on 10,000 bootstrap replicates. In that figure, the dashed blue lines represent 95 % confidence intervals for the parameters, none of which includes 0. There are hints in the figure that the distribution of the estimators (especially $\hat{\theta }_1$) may not be Normal. This is likely due to the constraint $\theta _3\le \theta _2\le \theta _1$. In the bottom row of Fig. 5, one can observe that parameters on the boundary of their domain are relatively frequent: $\hat{\theta }_1=\hat{\theta }_2$ in 14.3 % of the replicates, $\hat{\theta }_3=\hat{\theta }_2$ in 9.9 % of the replicates, and $\hat{\theta }_1=\hat{\theta }_2=\hat{\theta }_3$ in 4.8 % of the replicates.

To check for model adequacy, a random sample of size 500 from the fitted model was generated. A test of the hypothesis that the underlying copula of this sample is the same as that of the original data was then carried out using the rank-based procedure in [32]. The test statistic was computed with the R package TwoCop and led to an approximate *p*-value of 31 %, suggesting that the fit is not inadequate.

**Fig. 6:**
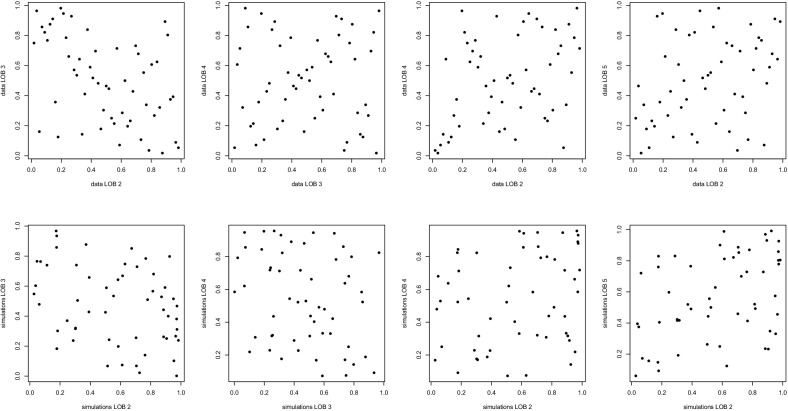
Adequacy check for the fully nested Archimedean copula model: ranks of pairs of residuals (*top row*) and pairs of simulations from the model (*bottom row*)

As an additional informal check, random samples of size 55 were drawn from the fitted 6-dimensional copula and compared visually to the empirical copula by looking at rank plots of selected pairs. Figure 6 shows one result from such a comparison of pairs (LOB 2, LOB $\ell$) with $\ell \in \{ 3,4,5\}$ and (LOB 3, LOB 4). The rank plots derived from the residuals are in the top row, and those corresponding to the random sample are in the bottom row. The positive dependence between Ontario risks seems to be accurately captured by the model. Although the negative association between LOBs 2 and 3 is taken into account, one can see in the second column of Fig. 6 that negative dependence is induced between LOBs 3 and 4. This is an artifact of the dependence structure, which assumes from the start that the pairs $(-3,\ell )$, with $\ell \in \{ 2,4,5\}$ have the same degree of association. Table 4 suggests that this is not the case. This issue could have been avoided by grouping LOB 2 and LOB 3 earlier in the structure, but at the expense of the overall fit of the model. A more flexible modeling approach is presented below.

## Copula-based risk aggregation model

In this section, a hierarchical approach to loss triangle modeling is considered. It appears to have been originally proposed by Swiss reinsurance practitioners [9, 35] but was formalized in [4]. Estimation and validation procedures for this class of models are described in [10, 11], where rank-based clustering techniques are also proposed for selecting an appropriate structure.

**Fig. 7:**
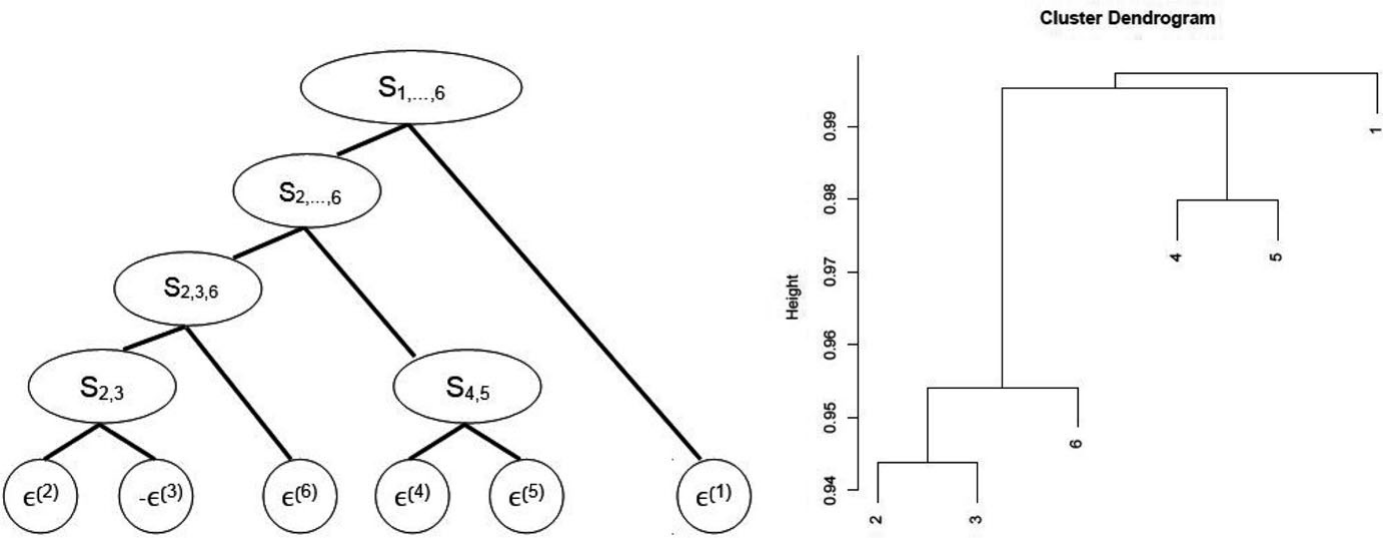
Illustration of the tree structure and dendrogram for the copula-based aggregation model

The model is defined using a tree comprising $d-1$ nodes, each of which has two branches. An example of such a structure is shown in the left panel of Fig. 7. At each node, a copula describes the dependence between the two components which are then summed and viewed as a single risk in higher levels of the hierarchy. For example, $C_{4,5}$ denotes the copula linking $\varepsilon ^{(4)}$ and $\varepsilon ^{(5)}$ and $S_{4,5}=\varepsilon ^{(4)}+\varepsilon ^{(5)}$, while $C_{2,\ldots ,6}$ is the copula linking aggregated risks $S_{2,3,6}$ and $S_{4,5}$.

A joint distribution for the *d* variables is then defined in terms of $d-1$ bivariate copulas and *d* marginal distributions under a conditional independence assumption. This assumption, which is reasonable in the present context, states that conditional on a sum at a given node, the descendents of that node are independent of the non-descendents. For additional details, see [4, 11].

This strategy is simple to implement, as it builds on tools already available for bivariate copula selection, inference, and validation. Furthermore, the $d-1$ copulas in the model can be chosen freely, thereby providing great flexibility in the dependence structure. Moreover, hierarchical clustering techniques can be adapted to obtain an appropriate tree structure.

As explained in [11], it is appealing to model first the risks that are the most dependent in some sense. In this paper, the distance based on Kendall’s $\tau$,

$$\begin{aligned} \Delta (\varepsilon ^{(\ell )},\varepsilon ^{(k)})=\sqrt{1-\tau ^2(\varepsilon ^{(\ell )},\varepsilon ^{(k)})}, \end{aligned}$$

is maximized at each step to obtain the dendrogram displayed in the right panel of Fig. 7. Risks 2 and 3 are grouped in the first step. Given that they are negatively associated, it was deemed preferable to work with $-\varepsilon ^{(3)}$ as was done in the previous section.

**Table 6:** Results of tests of independence at each aggregation step

Variables		$\tau$	*p*-value	
			Van der Waerden test	Kendall test
$\varepsilon ^{(2)}$	$-\varepsilon ^{(3)}$	0.331	0.0004	0.0004
$S_{2,3}$	$\varepsilon ^{(6)}$	0.300	0.0020	0.0012
$\varepsilon ^{(4)}$	$\varepsilon ^{(5)}$	0.200	0.0541	0.0311
$S_{2,3,6}$	$S_{4,5}$	0.098	0.0406	0.2925
$S_{2,\ldots ,6}$	$\varepsilon ^{(1)}$	0.075	0.3401	0.4204

Before selecting appropriate copulas for each aggregation step, Kendall and van der Waerden tests of independence were performed to see if the dependence is significant. The resulting *p*-values are shown in Table 6, where one can see that independence is rejected for the first four aggregation steps, but not at the last one. This is not surprising as the preliminary analysis of the data already suggested that the Atlantic BI line of business is not related to the others. Unlike the nested Archimedean copula model, the risk aggregation model captures the existing dependence between West BI and country-wide Liability lines, and includes the latter in the dependence analysis.

Given that the independence hypothesis cannot be rejected at the last node, there are only four copulas to be fitted, namely $C_{2,3}$, $C_{2,3,6}$, $C_{4,5}$ and $C_{2,\ldots ,6}$. Based on rank plots, tests of extremeness from [6] and goodness-of-fit tests based on the Cramér–von Mises distance $S_n$, parametric families of bivariate copulas were selected and fitted by maximum pseudo-likelihood. The final choices are summarized in Table 7.

**Table 7:** Copula family and parameter estimates

Step	Copula	Parameter	SD	Kendall’s $\tau$	*p*-value GoF test
$C_{2,3}$	Plackett	5.349	2.021	0.36	0.523
$C_{2,3,6}$	Frank	2.864	0.986	0.29	0.714
$C_{4,5}$	Clayton	0.548	0.215	0.22	0.147
$C_{2,\ldots ,6}$	$t_2$	0.162	0.180	0.10	0.358

The model validation technique described in [11] was used. It relies on a simulation algorithm proposed in [4] and validated in [25]. Based on a random sample of size 500 from the model, the test in [32] led to an approximate *p*-value of 52 %. Therefore, the null hypothesis that both samples are coming from the same copula cannot be rejected. This suggests that the selected hierarchical model is appropriate, and that the conditional independence assumption is reasonable. A visual check of the latter assumption confirms this finding.

**Fig. 8:**
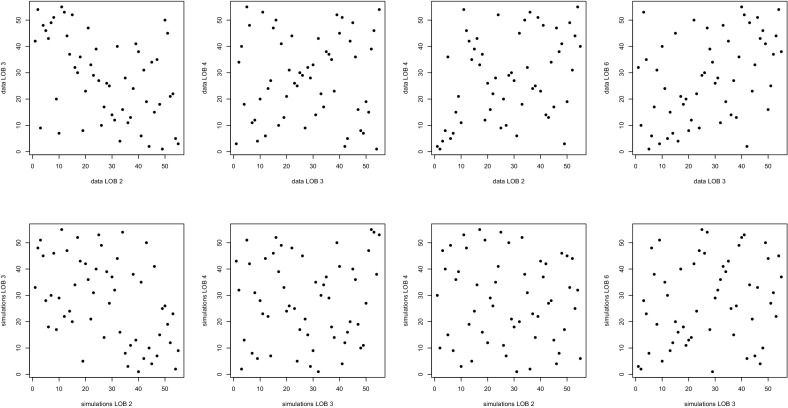
Adequacy check for the copula-based risk aggregation model: ranks of pairs of residuals (*top row*) and pairs of simulations from the model (*bottom row*)

Looking at Fig. 8, one can see that the pitfalls of the nested Archimedean copula model have been addressed: there is no negative dependence between LOBs 3 and 4, and the model induces positive dependence between LOBs 3 and 6. However, the extent of the association between Ontario AB and BI risks is not portrayed as vividly in the aggregation model as it was in the nested Archimedean copula model. Over all, the risk aggregation model provides a faithful description of the data.

Note that if desired, a modification of the tree structure would make it possible to account for the dependence between LOB 2 and the pair (LOB 4, LOB 5). In that case, however, the negative dependence between LOBs 2 and 3 would be masked.

## Predictive distribution and risk capital

The goal of loss triangle modeling is to forecast the unpaid loss by completing the triangle into a rectangle. Insurance companies are interested in the expected unpaid loss—the reserve—but also in its standard deviation, and other risk measures defined in terms of a risk tolerance $\kappa \in (0,1)$ such as the Value-at-Risk (VaR) and the Tail Value-at-Risk (TVaR). In principle, these various measures could all be computed for the nested Archimedean copula model (Model I) and the risk aggregation model (Model II), given that they both specify a distribution for the total unpaid claims. As these distributions cannot be obtained explicitly through a convolution, however, all risk measures must be estimated by simulation. To obtain one realization of the total unpaid loss, one can proceed as follows.

**Simulation procedure**

1. Simulate 45 observations from the dependence model.
2. Transform these observations into loss ratios $X_{ij}^{(\ell )}$ for each LOB $\ell \in \{1,\ldots ,6\}$, development year $j \in \{ 2,\ldots ,10\}$ and accident year $i \in \{ 12-j,\ldots ,10\}$ by using appropriate inverse probability transforms.
3. For each LOB $\ell \in \{ 1,\ldots ,6\}$, compute the simulated unpaid loss
  $$\begin{aligned} X^{(\ell )}=\sum _{i=2}^{10}\sum _{j=12-i}^{10}p_{i}^{(\ell )}X_{ij}^{(\ell )} \end{aligned}$$
  as well as the total unpaid loss $S=X^{(1)}+\cdots +X^{(6)}$.

Consistent estimates of the risk measures can be derived easily from *n* independent copies of the unpaid loss $S_1, \ldots , S_n$. Let $F_n$ be the corresponding empirical distribution function. Then

$$\begin{aligned} \widehat{\mathrm{VaR}}_{\kappa }(S) = \inf \{s \in \mathbb {R} |F_n (s)\ge \kappa \} = s_\kappa \end{aligned}$$

and

$$\begin{aligned} \widehat{\mathrm{TVaR}}_{\kappa }(S) = \frac{1}{1-\kappa } \left[ \frac{1}{n}\sum _{j=1}^n S_j\mathbf {1}(S_j> s_\kappa ) + s_\kappa \{F_n (s_\kappa )-\kappa \}\right] . \end{aligned}$$

**Table 8:** Risk measures for 500,000 simulations

Model	Average	SD	$$\mathrm{VaR}_{95~\%}$$	$$\mathrm{VaR}_{99~\%}$$	$$\mathrm{TVaR}_{99~\%}$$
I	$438,115	$13,706	$460,938	$470,750	$475,697
II	$438,101	$13,808	$461,179	$471,486	$476,763

Table 8 shows risk measures for the total unpaid loss based on 500,000 simulations for Models I and II. Given the GLMs fitted to the marginal distributions, one would expect an average total unpaid loss of $438,088; the small discrepancy between this value and the approximations is due to simulation. The risk measures are all smaller for Model I than for Model II. This is slightly surprising because Model II takes into account the negative dependence between LOBs 2 and 3; intuitively, one would thus expect more risk diversification under Model II than under Model I. Nevertheless, Model II is more conservative than Model I in the sense that it does not assume that LOB 6 is independent from the other lines of business. In addition, Model II is based in part on Plackett and $$t_2$$ copulas, which exhibit tail dependence, whereas members of Frank’s copula family in Model I do not.

**Table 9:** Risk capital allocation for 500,000 simulations

Model	$$\mathrm{TVaR}_{99~\%}$$-based capital allocations						Total
	LOB 1	LOB 2	LOB 3	LOB 4	LOB 5	LOB 6	
Silo	$42,510	$157,764	$87,141	$90,237	$22,027	$118,807	$518,485
I	$37,006	$151,247	$82,578	$74,320	$18,639	$111,907	$475,697
II	$36,891	$147,418	$79,719	$81,928	$19,285	$111,521	$476,763

Insurance companies also have to determine capital allocations, i.e., the share of the risk capital to be allocated to each LOB. This exercise helps to identity the most and least profitable sectors of activities in a company. Capital allocation principles have first been introduced in [38]; see [5] for a review. Here, TVaR-based capital allocations are used. If

$$\begin{aligned} X^{(\ell )}=\sum _{i=2}^{10}\sum _{j=12-i}^{10}p_{i}^{(\ell )}X_{ij}^{(\ell )} \end{aligned}$$

is the unpaid loss for LOB $\ell$, the capital allocated to that LOB is

$$\begin{aligned} \mathrm{TVaR}_{\kappa }(X^{(\ell )};S) = \frac{{\mathrm{E}[X^{(\ell )} \mathbf {1}\{S>\mathrm{VaR}_{\kappa }(S)\}] + \beta _\kappa \, \mathrm{E}[X^{(\ell )} \mathbf {1}\{S=\mathrm{VaR}_{\kappa }(S)\}]}}{1-\kappa }, \end{aligned}$$

where $\beta _\kappa = [F_{S}\{\mathrm{VaR}_{\kappa }(S)\}-\kappa ]/\Pr \{S=\mathrm{VaR}_{\kappa }(S)\}$ if the denominator is strictly positive and 0 otherwise. This quantity can be estimated by

$$\begin{aligned} \widehat{\mathrm{TVaR}}_{\kappa }(X^{(\ell )};S) = \frac{1}{n(1-\kappa )}\left\{ \sum _{j=1}^n X^{(\ell )}_{j}\mathbf {1} (S_j> s_\kappa ) + \frac{F_{n}(s_\kappa )-\kappa }{\displaystyle \frac{1}{n} \sum\nolimits_{k=1}^n \mathbf {1}(S_k = s_\kappa )} \sum _{j=1}^n X^{(\ell )}_{j}\mathbf {1}(S_j=s_\kappa )\right\} , \end{aligned}$$

where $X^{(\ell )}_{1},\ldots , X^{(\ell )}_{n}$ are the *n* realizations of $X^{(\ell )}$ corresponding to the realizations $S_1,\ldots ,S_n$.

In Table 9, TVaR-based capital allocations are shown for both models as well as for the “Silo” method, which is widespread in industry [2]. It is clear that the Silo method overestimates the total capital required as it implicitly assumes that risks are comonotonic, thereby preventing any form of diversification. The results for Models I and II are similar. While the capital allocations for LOBs 4 and 5 are higher in Model II than in Model I, they are lower for LOBs 2 and 3, outlining the additional risk diversification that is possible in the presence of negative dependence.

The risk measures in Tables 8 and 9 could be used to set internal capital targets, but they do not incorporate parameter uncertainty, as the model is assumed to be correct. However, a parametric bootstrap can be used in order to quantify estimation error and to tackle potential model over-fitting; see, e.g., [37] or [39]. For the present purpose, it was assumed that the tree structure, the copula families, and the marginal distributions are given, except for their parameter values. The following procedure was then repeated a large number of times (10,000 times here) in order to obtain the approximate distribution of the unpaid loss, including parameter uncertainty.

**Parametric bootstrap procedure**

1. Simulate 55 observations from the dependence model, and transform them into observations of the loss ratios for the top triangle, i.e., all accident years $i \in \{1,\ldots ,10\}$ and development years $j \in \{1,\ldots ,11-i\}$, using the inverse marginal distributions.
2. Fit the marginal GLMs (log-normal for LOB 1 and Gamma for LOBs 2–6).
3. Compute the residuals from the GLMs.
4. Fit the copula model to the ranks of the residuals obtained.
5. From this new model, simulate the total unpaid loss using the steps described under “Simulation procedure”. The aggregate value is the simulated total unpaid loss.

The results for the nested Archimedean copula model should be interpreted with caution, however, because the constraints on the dependence parameters in this model, and notably the fact that $\hat{\theta }_2$ is close to $\hat{\theta }_1$, may invalidate the parametric bootstrap [3].

**Fig. 9:**
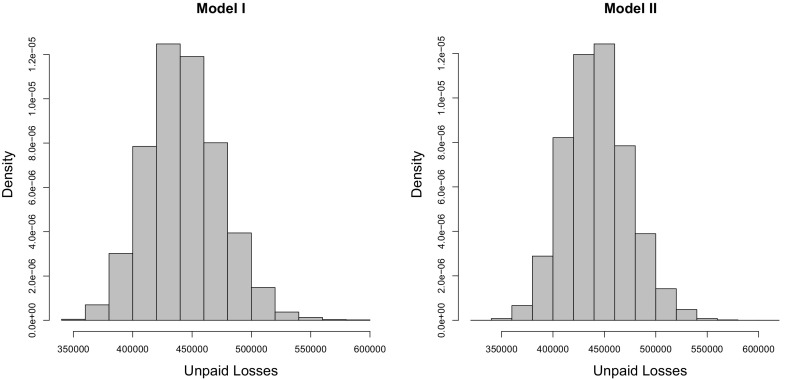
Predictive distributions based on 10,000 bootstrap replicates

Tables 10 and 11 show risk measures and capital allocations obtained with 10,000 bootstrap simulations, while Fig. 9 shows the predictive distribution obtained for Model I (left) and Model II (right). The risk measures in Table 10 are similar for both models and are much higher than those reported in Table 8; this highlights the importance of incorporating parameter uncertainty. Unsurprisingly, most of the increase in risk measures when including parameter uncertainty is due to the $6\times 20=120$ marginal GLM parameters. Table 12 shows the risk measures obtained with the parametric bootstrap procedure without Step 4, i.e., the dependence parameters are fixed to their initial value estimated with the original data. The resulting risk measures are close to those found in Table 10, even though the uncertainty in the copula parameters is not accounted for when Step 4 is omitted.

**Table 10:** Risk measures for 10,000 bootstrap simulations

Model	Average	SD	$$\mathrm{VaR}_{95~\%}$$	$$\mathrm{VaR}_{99~\%}$$	$$\mathrm{TVaR}_{99~\%}$$
I	$443,041	$31,291	$496,780	$521,293	$539,205
II	$442,957	$31,038	$496,470	$522,417	$535,536

**Table 11:** Risk capital allocation for 10,000 bootstrap simulations

Model	$$\mathrm{TVaR}_{99~\%}$$-based capital allocations						Total
	LOB 1	LOB 2	LOB 3	LOB 4	LOB 5	LOB 6	
Silo	$60,740	$189,466	$103,465	$111,946	$26,637	$157,345	$649,599
I	$40,519	$167,492	$90,228	$75,015	$18,565	$147,386	$539,205
II	$41,919	$158,306	$83,978	$88,665	$20,858	$141,810	$535,536

**Table 12:** Risk measures for 10,000 bootstrap simulations including uncertainty for marginal parameters only

Model	Average	SD	$$\mathrm{VaR}_{95~\%}$$	$$\mathrm{VaR}_{99~\%}$$	$$\mathrm{TVaR}_{99~\%}$$
I	$443,554	$31,390	$496,781	$522,696	$535,069
II	$442,937	$30,928	$495,620	$520,986	$534,703

Finally, the figures in Table 11 are in line with those of Table 9. In particular, observe that Model II allocates less capital to LOB 6 than Model I, reflecting the fact that LOB 6 is related to LOBs 2 and 3 in Model II. In view of these results, the insurer might consider increasing the volume of LOB 3 to take better advantage of risk diversification.

## Summary and discussion

In this paper, rank-based procedures were introduced for the selection, estimation and validation of dependence structures for run-off triangles of property and casualty insurance claim data. The approach was illustrated using data from six lines of business of a large Canadian insurance company. Two hierarchical approaches were considered for modeling the pairwise dependence between different lines of business, i.e., fully nested Archimedean copulas and a copula-based risk aggregation model.

As simple and convenient as the nested Archimedean copula model may seem, its implementation raises more issues than one might anticipate initially. The success of this approach hinges on the choice of hierarchy and Archimedean generators at each of its levels. In principle, different Archimedean generators could be used throughout the structure, but the conditions required to ensure that the construction is valid are not always easy to verify. As there is no selection technique for generators, practitioners typically assume that they are all from the same parametric family $\varphi _\theta$. In the latter case, conditions for the validity of the nested copula typically boil down to the constraint $\theta _1\ge \cdots \ge \theta _d$; see, e.g., [20].

As illustrated in the present paper, the use of the same generator throughout a fully nested Archimedean copula model has strong implications on the dependence structure. In particular, each variable is linked by the same bivariate copula to any variable appearing in a lower level of the hierarchy and, therefore, shares the same dependence characteristics with all of them in terms of symmetry, tail dependence, etc. In addition, the conditions stated in Eq. (1) are not only restrictive, but are also problematic for the parametric bootstrap. Indeed, when a bootstrap sample leads to unconstrained estimates $\hat{\theta }_1,\ldots ,\hat{\theta }_d$ such that $\hat{\theta }_1\ge \cdots \ge \hat{\theta }_d$ fails, one or more of the constrained parameter estimates end up being equal to 0. When this happens repeatedly, the dependence between the LOBs is underestimated. Thus, it is still unclear that this model can be used in a parametric bootstrap procedure to obtain the predictive distribution of unpaid losses, due to the optimization problem that is not standard.

Working with the risk aggregation model allows one to avoid most of these issues. The tree structure can be determined using hierarchical clustering and the copulas can be chosen freely at each aggregation step. In addition, standard tools for bivariate copula selection, estimation, and validation are available. Moreover, the application of the parametric bootstrap to this context is standard, as there are no constraints on the parameters. Overall, the model provides greater flexibility and the dependence structure can be considerably more complex than what can be achieved with the nested Archimedean approach. However, the conditional independence assumption must be satisfied (at least approximately) and formal tools for checking this assumption remain to be developed. Another minor irritant is the fact that simulation from this model relies on the Iman–Conover reordering algorithm, which is efficient but not yet included in standard software; in contrast, sampling from the fully nested Archimedean copula is easily done with the R package copula.

Perhaps the most significant limitation of the rank-based approach to risk aggregation modeling described here is that it can only be applied to data or residuals that are (at least approximately) identically distributed. Another requirement for this approach to make sense is that the sums that are linked by the copulas have the same number of components. This means that the risk aggregation model cannot be extended easily to include calendar year dependence, as Abdallah et al. [1] did using nested Archimedean copulas. Unfortunately, this approach is not amenable to estimation and validation procedures based on ranks, as there is then only one observation for each copula in the model.

## Appendix 1: Nested Archimedean copula density

The 3-dimensional fully nested Archimedean copula is defined, for all $u, v, w \in (0,1)$, by

$$\begin{aligned} C(u,v,w)=C_{\theta _2} \{ w,C_{\theta _1}(u,v)\}, \end{aligned}$$

where $\theta _1 \ge \theta _2 \ge 0$. To ease notation, let $C^{(i,j)}_\theta (u,v) = \partial ^{i+j}C_\theta (u,v)/\partial u^i\partial v^j$ for $i,j\in \{0,1,2\}$. The density of the nested Archimedean copula can be derived easily using the chain rule, viz.

$$\begin{aligned} c(u,v,w)&=\frac{\partial ^3}{\partial u\partial v\partial w} \, C_{\theta _2} \{ w,C_{\theta _1}(u,v)\} =\frac{\partial ^2}{\partial u\partial v} \, C^{(1,0)}_{\theta _2} \{ w,C_{\theta _1}(u,v)\} \\&=\frac{\partial }{\partial u} \left[ C^{(1,1)}_{\theta _2} \{ w,C_{\theta _1}(u,v)\} C^{(0,1)}_{\theta _1}(u,v)\right] \\&=C^{(1,2)}_{\theta _2} \{ w,C_{\theta _1}(u,v)\} C^{(1,0)}_{\theta _1}(u,v)C^{(0,1)}_{\theta _1}(u,v)+ C^{(1,1)}_{\theta _2}\{ w,C_{\theta _1}(u,v)\} C^{(1,1)}_{\theta _1}(u,v). \end{aligned}$$

This expression is explicit, though it involves partial derivatives. In the case of the Frank family, the expressions required are the copula

$$\begin{aligned} C_\theta (u,v)=-\frac{1}{\theta }\ln \left\{ 1+\frac{(e^{-\theta u}-1)(e^{-\theta v}-1)}{(e^{-\theta }-1)}\right\} , \end{aligned}$$

its density

$$\begin{aligned} C^{(1,1)}_\theta (u,v)=c_\theta (u,v) = \frac{-\theta e^{-\theta (u+v)}(e^{-\theta }-1)}{\{(e^{-\theta }-1)+(e^{-\theta u}-1)(e^{-\theta v}-1)\}^2}, \end{aligned}$$

and the following partial derivatives:

$$\begin{aligned} C^{(1,0)}_\theta (u,v)&=\frac{\partial C_\theta (u,v)}{\partial u}=\frac{ e^{-\theta u}(e^{-\theta v}-1)}{(e^{-\theta }-1)+(e^{-\theta u}-1)(e^{-\theta v}-1)} = C^{(0,1)}_\theta (v,u),\\ C^{(1,2)}_\theta (u,v)&=\frac{\partial c_\theta (u,v)}{\partial v} = \frac{-\theta ^2(e^{-\theta }-1)e^{-\theta (u+v)}\{(e^{-\theta v}+1)(e^{-\theta u}-1)-(e^{-\theta }-1)\}}{ \{ (e^{-\theta }-1)+(e^{-\theta u}-1)(e^{-\theta v}-1)\}^3}. \end{aligned}$$

A similar procedure can be used to obtain the copula density in dimensions 4 and 5. The formulas are available from the authors upon request or can be derived through long but routine calculations facilitated by resorting to a symbolic calculator such as Maple or Mathematica.

## Appendix 2: Data and marginals

Tables 13, 14, 15, 16, 17 and 18 provide the net earned premiums and the cumulative paid losses for accident years 2003–12 inclusively for each of LOBs 1–6 developed over at most 10 years. To preserve confidentiality, all figures were multiplied by a constant.

**Table 13:** Cumulative paid losses for LOB 1

Accident year	Development lag (in months)										
	12	24	36	48	60	72	84	96	108	120	Premiums
2003	1404	4445	8037	9885	11,272	12,465	13,892	14,433	15,127	15,580	43,028
2004	437	2222	3805	4821	6065	6961	7266	8385	8645		29,905
2005	408	2170	4369	6995	7996	9450	11,104	11,569			31,780
2006	372	1785	4757	6368	8377	9470	10,122				30,381
2007	404	1965	3953	6454	7507	8142					28,939
2008	355	2069	3661	5161	6121						27,844
2009	1316	2955	4839	5896							25,812
2010	298	2595	4582								24,188
2011	402	2475									23,412
2012	553										23,993

**Table 14:** Cumulative paid losses for LOB 2

Accident year	Development lag (in months)										
	12	24	36	48	60	72	84	96	108	120	Premiums
2003	3488	14,559	27,249	37,979	49,561	55,957	58,406	60,862	63,280	63,864	85,421
2004	1169	12,781	20,550	31,547	42,808	47,385	50,251	50,978	51,272		98,579
2005	1478	10,788	25,499	34,279	43,057	49,360	52,329	52,544			103,062
2006	1186	11,852	22,913	32,537	41,824	48,005	52,542				108,412
2007	1737	13,881	25,521	38,037	43,684	47,755					111,176
2008	1571	12,153	27,329	41,832	51,779						112,050
2009	1199	17,077	29,876	44,149							112,577
2010	1263	16,073	28,249								113,707
2011	986	10,003									126,442
2012	683										130,484

**Table 15:** Cumulative paid losses for LOB 3

Accident year	Development lag (in months)										
	12	24	36	48	60	72	84	96	108	120	Premiums
2003	2279	8683	15,136	21,603	27,650	30,428	32,004	32,592	33,009	34,140	76,620
2004	2139	7077	13,159	16,435	20,416	22,598	24,171	25,034	25,714		65,691
2005	1420	4888	8762	12,184	14,482	15,633	17,089	17,710			55,453
2006	1510	5027	10,763	15,799	19,269	22,504	24,807				54,006
2007	1693	5175	8216	12,263	16,918	20,792					55,425
2008	2097	7509	10,810	15,673	19,791						59,100
2009	2094	5174	8062	12,389							54,438
2010	1487	4789	7448								53,483
2011	1868	6196									52,978
2012	2080										57,879

**Table 16:** Cumulative paid losses for LOB 4

Accident year	Development lag (in months)										
	12	24	36	48	60	72	84	96	108	120	Premiums
2003	13,714	24,996	31,253	38,352	44,185	46,258	47,019	47,894	48,334	48,902	116,491
2004	6883	16,525	24,796	29,263	32,619	33,383	34,815	35,569	35,612		111,467
2005	7933	22,067	32,801	38,028	44,274	44,948	46,507	46,665			107,241
2006	7052	18,166	25,589	31,976	36,092	38,720	39,914				105,687
2007	10,463	23,982	31,621	36,039	38,070	41,260					105,923
2008	9697	28,878	41,678	47,135	50,788						111,487
2009	11,387	37,333	48,452	55,757							113,268
2010	12,150	32,250	40,677								121,606
2011	5348	14,357									110,610
2012	4612										104,304

**Table 17:** Cumulative paid losses for LOB 5

Accident year	Development lag (in months)										
	12	24	36	48	60	72	84	96	108	120	Premiums
2003	3043	5656	7505	8593	9403	10,380	10,450	10,812	10,856	10,860	116,491
2004	2070	4662	6690	8253	9286	9724	9942	10,086	10,121		111,467
2005	2001	4825	7344	8918	9824	10,274	10,934	11,155			107,241
2006	1833	4953	7737	9524	10,986	11,267	11,579				105,687
2007	2217	5570	7898	8885	9424	10,402					105,923
2008	2076	5681	8577	10,237	12,934						111,487
2009	2025	6225	9027	10,945							113,268
2010	2024	5888	8196								121,606
2011	1311	3780									110,610
2012	912										104,304

**Table 18:** Cumulative paid losses for LOB 6

Accident year	Development lag (in months)										
	12	24	36	48	60	72	84	96	108	120	Premiums
2003	4157	9558	13,131	17,460	19,608	21,124	21,900	23,360	23,377	23,575	55,484
2004	4158	9956	14,860	18,024	20,397	22,068	23,312	24,555	25,137		65,705
2005	3989	10,519	15,877	20,274	23,428	26,495	30,974	31,580			73,879
2006	4012	10,904	16,141	19,643	21,954	26,215	28,095				91,473
2007	4322	10,814	16,086	20,186	24,157	27,222					87,212
2008	6379	14,524	19,058	24,108	28,329						89,455
2009	5291	14,620	20,799	25,131							90,341
2010	4946	12,956	18,007								89,212
2011	5674	15,026									91,606
2012	5478										99,982

**Table 19:** Parameter and reserve estimations

LOB $\ell$	1	2	3	4	5	6
GLM	Log-normal	Gamma	Gamma	Gamma	Gamma	Gamma
$\zeta ^{(\ell )}$	−4.031 (0.157)	−3.628 (0.148)	−3.501 (0.098)	−2.365 (0.173)	−4.064 (0.148)	−2.872 (0.167)
Accident year						
2	−0.226 (0.153)	−0.750 (0.151)	0.053 (0.097)	−0.413 (0.174)	−0.121 (0.151)	0.101 (0.177)
3	0.022 (0.161)	−0.729 (0.160)	−0.156 (0.100)	−0.196 (0.183)	0.171 (0.161)	0.163 (0.177)
4	−0.028 (0.168)	−0.651 (0.168)	0.239 (0.105)	−0.112 (0.190)	0.129 (0.168)	−0.136 (0.184)
5	−0.112 (0.177)	−0.741 (0.174)	0.137 (0.110)	−0.095 (0.199)	0.092 (0.173)	−0.024 (0.191)
6	−0.183 (0.189)	−0.574 (0.185)	0.120 (0.117)	−0.001 (0.210)	0.396 (0.187)	0.095 (0.203)
7	0.170 (0.205)	−0.574 (0.200)	0.003 (0.127)	0.197 (0.227)	0.254 (0.200)	0.069 (0.219)
8	0.032 (0.228)	−0.658 (0.220)	−0.160 (0.141)	−0.012 (0.253)	0.055 (0.222)	−0.017 (0.246)
9	0.131 (0.268)	−1.147 (0.255)	0.169 (0.167)	−0.628 (0.295)	−0.259 (0.260)	0.131 (0.289)
10	0.261 (0.362)	−1.625 (0.340)	0.175 (0.226)	−0.754 (0.393)	−0.676 (0.348)	−0.032 (0.390)
Dev. lag						
2	1.311 (0.154)	2.061 (0.145)	0.815 (0.096)	0.450 (0.167)	0.419 (0.149)	0.420 (0.167)
3	1.438 (0.161)	2.065 (0.151)	0.817 (0.101)	−0.055 (0.175)	0.114 (0.155)	0.076 (0.174)
4	1.150 (0.168)	2.018 (0.158)	0.849 (0.106)	−0.507 (0.183)	−0.358 (0.163)	−0.095 (0.182)
5	0.874 (0.177)	1.818 (0.166)	0.717 (0.112)	−0.759 (0.193)	−0.582 (0.173)	−0.406 (0.192)
6	0.636 (0.189)	1.297 (0.176)	0.283 (0.120)	−1.580 (0.207)	−1.154 (0.182)	−0.481 (0.206)
7	0.392 (0.205)	0.773 (0.193)	−0.115 (0.129)	−1.899 (0.223)	−1.870 (0.201)	−0.757 (0.226)
8	0.137 (0.228)	−0.493 (0.216)	−1.001 (0.143)	−2.670 (0.250)	−2.103 (0.219)	−1.215 (0.248)
9	−0.291 (0.268)	−0.429 (0.255)	−1.375 (0.169)	−3.762 (0.298)	−3.849 (0.257)	−2.612 (0.304)
10	−0.522 (0.362)	−1.358 (0.340)	−0.715 (0.226)	−2.960 (0.393)	−6.248 (0.348)	−2.764 (0.390)
sd or scale	0.326 (0.031)	10.700 (2.009)	24.046 (4.554)	8.038 (1.502)	10.078 (1.891)	8.021 (1.499)
Reserve	36,063	132,919	78,665	73,220	18,290	98,931
C-L reserve	35,411	146,794	76,500	75,551	18,726	100,704
